# Linalool attenuated ischemic injury in PC12 cells through inhibition of caspase‐3 and caspase‐9 during apoptosis

**DOI:** 10.1002/fsn3.3057

**Published:** 2022-09-20

**Authors:** Azar Hosseini, Elham Pourheidar, Arezoo Rajabian, Elham Asadpour, Hossein Hosseinzadeh, Hamid Reza Sadeghnia

**Affiliations:** ^1^ Pharmacological Research Center of Medicinal Plants Mashhad University of Medical Sciences Mashhad Iran; ^2^ Department of Pharmacology Faculty of Medicine, Mashhad University of Medical Sciences Mashhad Iran; ^3^ Department of Intensive Care Unit Hazrat Rasul akram Hospital Iran University of Medical Sciences Tehran Iran; ^4^ Department of Internal Medicine, Faculty of Medicine Mashhad University of Medical Sciences Mashhad Iran; ^5^ Anesthesiology and Critical Care Research Center Shiraz University of Medical Sciences Shiraz Iran; ^6^ Pharmaceutical Research Center Pharmaceutical Technology Institute Mashhad University of Medical Sciences Mashhad Iran; ^7^ Division of Neurocognitive Sciences, Psychiatry and Behavioral Sciences Research Center Mashhad University of Medical Sciences Mashhad Iran

**Keywords:** (±) linalool, (*R*)‐(−) linalool, ischemia, oxygen–glucose deprivation/reoxygenation

## Abstract

Numerous studies have indicated the pharmacological properties of linalool, a volatile terpene alcohol found in many flowers and spice plants, including anti‐nociceptive, anti‐inflammatory, and neuroprotective activities. The aim of this study was to explore the mechanisms of neuroprotection provided by (±) linalool and its enantiomer, (*R)*‐(−) linalool against oxygen, and glucose deprivation/reoxygenation (OGD/R) in PC12 cells. PC12 cells were treated with (±) linalool and (*R)*‐(−) linalool before exposure to OGD/R condition. Cell viability, reactive oxygen species (ROS) production, malondialdehyde (MDA) level, DNA damage, and the levels of proteins related to apoptosis were evaluated using MTT, comet assay, and western blot analysis, respectively. IC_50_ values for the PC12 cells incubated with (±) linalool and *(R)*‐(−) linalool were 2700 and 2600 μM after 14 h, as well as 5440 and 3040 μM after 18 h, respectively. Survival of the ischemic cells pre‐incubated with (±) linalool and (*R)*‐(−) linalool (100 μM of both) increased compared to the cells subjected to the OGD/R alone (*p* < .001). ROS and MDA formation were also decreased following incubation with (±) linalool and *(R)*‐(−) linalool compared to the OGD/R group (*p* < .01). In the same way, pre‐treatment with (±) linalool and *(R)*‐(−) linalool significantly reduced OGD/R‐induced DNA injury compared to that seen in OGD/R group (*p* < .001). (±) Linalool and *(R)*‐(−) linalool also restored Bax/Bcl‐2 ratio and cleaved caspase‐3 and caspase‐9 (*p* < .001, *p* < .01) following ischemic injury. The neuroprotective effect of linalool against ischemic insult might be mediated by alleviation of oxidative stress and apoptosis.

## Graphical abstract

This study indicated, for the first time, that (±) linalool and its enantiomer, (R)‐(‐) linalool protected PC12 cells against injury induced by oxygen and glucose deprivation/reoxygenation (OGD/R) via the mitigation of oxidative stress and the downregulation of Bax/Bcl‐2, caspase‐3 and caspase‐9, resulting in decreased apoptotic cell death.

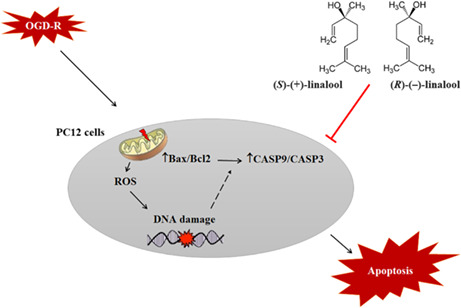

## 1 INTRODUCTION

Lack of glucose and oxygen supply to an area of brain due to decreased blood flow to the brain's tissue causes ischemic strokes, the most prevalence form of strokes (Chiu et al., 2014). Considering high percentage of mortality and disability due to stroke (Chen et al., 2017), as well as limitation in the current treatments, recent studies focus on promising strategies for prevention and treatment of ischemic cerebral injury (Page et al., 2012). A number of critical pathological processes including excitotoxicity, oxidative stress, and inflammation have been proposed to be involved in the cerebral ischemia (Lee et al., 2014). These alterations trigger death signaling pathways inducing cell apoptosis (Rodrigo et al., 2013).

3,7‐dimethylocta‐1,6‐dien‐3‐ol, also named as linalool, is a volatile terpene alcohol found in many flowers and spice plants (Stashenko & Martínez, 2008). Two enantiomeric forms (*R*)‐(−) linalool (licareol) and (*S*)‐(+)‐linalool (coriandrol) have been detected in the essential oils of aromatic plant species, including most teas such as black tea, green tea and honeybush tea (Casabianca et al., 1998; Sugawara et al., 1998; Zhao et al., 2021). Traditionally, the plant species containing linalool have been used in a number of acute and chronic ailments (Altınok‐Yipel et al., 2019; Batista et al., 2010; Elisabetsky et al., 1995; Letizia et al., 2003) including anxiety, depression, and convulsion (Maia et al., 2003; Maia & Mourão, 2016; Tucker et al., 2001). Recently, the anti‐depressant properties (dos Santos et al., 2018; Guzmán‐Gutiérrez et al., 2012, 2015), as well as the anxiolytic, antinociceptive, and anticonvulsant activities of linalool‐rich essential oils and linalool (Almeida et al., 2009; de Sousa et al., 2010; Souto‐Maior et al., 2017) have been reported.

Linalool has been shown to reduce the neuropathic pain via interaction with NMDA (N‐methyl‐D‐aspartate) receptors and suppression of the pro‐inflammatory cytokines (Peana et al., 2004; Pereira et al., 2018). Also, the anti‐depression and anxiolytic effects have been mediated through modulating monoaminergic system and antagonizing NMDA receptors (Pereira et al., 2018). Linalool also alleviated learning and spatial memory deficits, reduced β‐amyloid plaques, tauopathy, and pro‐inflammatory mediators (p38 MAPK, NOS2, COX2, and IL‐1b) in the hippocampus and amygdala of D‐galactose and aluminum trichloride‐induced Alzheimer's disease mouse model (Xu et al., 2017a). Linalool was found to induce neuroprotective effects through suppression of ROS, restoring antioxidant enzymes, as well as inhibition of microglia migration (Li et al., 2015). Moreover, linalool could protect neuronal cells from apoptotic cell death following ischemic/hypoxic injury (Park et al., 2016). Activation of nuclear factor‐erythroid 2‐related factor 2 (Nrf2) and heme oxygenase‐1 (HO‐1) pathway by *(R)*‐(−) linalool could protect against oxidative injury (Li et al., 2015; Xu et al., 2017a, 2017b).

This study was aimed to evaluate the neuroprotective and anti‐apoptotic effects of both *(R)*‐(−) linalool and (±) linalool in PC12 cells subjected to oxygen and glucose deprivation/reoxygenation (OGD/R), as a model of cerebral ischemia–reperfusion injury.

## 2 MATERIALS AND METHODS

### 2.1 Chemicals and reagents

The following chemicals and reagents were used in this study: (*R*)‐(−) linalool and (±) linalool, propidium iodide (PI), 2′,7′‐ dichlorofluorescin diacetate (DCFH_2_‐DA), protease inhibitor cocktail, bicinchoninic acid (BCA) protein assay kit, low melting point (LMP), and normal melting point (NMP) agarose (Sigma). Disodium salt of ethylene diaminetetraacetic acid (Na_2_EDTA), ethidium bromide, triton X‐ 100, 3‐(4, 5‐dimethylthiazol‐2‐yl)‐2,5‐diphenyltetrazolium bromide (MTT), tris(hydroxymethyl) aminomethane, dimethyl sulfoxide (DMSO) (Merck, Darmstadt, Germany). Penicillin and streptomycin, fetal bovine serum (FBS), high glucose Dulbecco's Modified Eagles Medium (DMEM, 4.5 g/L) (Gibco, Carlsbad, USA). Anti‐β‐actin (#4967), B‐cell lymphoma protein 2 (Bcl‐2)‐associated X (Bax, #2772), Bcl‐2 (#2870), cysteine‐aspartic acid protease‐3 (caspase‐3, #9664), caspase‐9 (#9507), and goat anti‐rabbit immunoglobulin G conjugated to horseradish peroxidase (#7074) antibodies (Cell Signaling Technology).

### 2.2 Cell culture and treatments

The PC12 cells were cultured in high glucose DMEM (containing 4500 mg/L of glucose) which was supplemented with 10% FBS and 1% antibiotics (penicillin/streptomycin) in a chamber with a humidified atmosphere of 5% CO_2_, at 37 °C. The cells were cultured in a 96‐well or 6‐well culture plates (at a density of 10^4^ and 10^6^ cell/well, respectively). Thereafter, the culture media of each well were replaced with fresh media containing 1, 1.5, 3, 6, 12.5, 25, 50, 100, 200, 800, 1600, 2000, 2500, and 3200 μM of *(R)*‐(−) linalool and (±) linalool. After 2 or 6 h pre‐treatment with linalool, the cells were exposed to OGD/R condition (an in vitro model of cerebral ischemia and reperfusion injury).

To produce OGD/R condition, the PC12 cell were placed in an anaerobic incubator (containing 1% O_2_, 94% N_2_, and 5% CO_2_) with the glucose‐free DMEM medium at 37°C. After 12 h, the cells were then returned to a normoxic incubator (95% air and 5% CO_2_) with normal glucose medium for 12 h. Finally, the cells were harvested for the following experiments (Forouzanfar et al., 2021).

### 2.3 Cell viability determination

At the end of treatment period, PC12 cell viability was determined using the MTT colorimetric method (Sadeghnia et al., 2014). One hundred microliters of MTT solution (5 mg/mL) was transferred to each well and incubated for 3 h at 37°C. After adding DMSO (150 μl), the absorbance of soluble formazan was measured with a microplate reader at 450–630 nm.

In addition, the half maximal inhibitory concentrations (IC_50_) of *(R)*‐(−) linalool and (±) linalool were estimated according to the viability of PC12 cells.

### 2.4 Intracellular ROS determination

Briefly, the cultured PC12 cells were pre‐incubated with 2′,7′‐ dichlorodihydrofluorescin diacetate (DCFH‐DA, 25 μM) at 37°C for 30 min to load the fluorescent dye. After washing the monolayer cells with PBS, they were treated with varying concentrations of (*R*)‐(−) linalool and (±) linalool and (1–3200 μM) for 2 or 6 h prior to OGD exposure (Wu & Yotnda, 2011). The results were presented as percentage of control (untreated group).

### 2.5 Determination of MDA formation

Malondialdehyde production, a marker of lipid peroxidation, was measured based on thiobarbituric acid‐reactive substances, as previously described (Hamid Reza Sadeghnia et al., 2017). At the end of treatments, a cell lysate was prepared by scraping the cell monolayers with trichloroacetic solution (1 ml, 2.5%). The homogenate was centrifuged (4000 *g*, 10 min, 4°C) and thiobarbituric acid (0.67% w/v, 0.8 ml) and trichloroacetic acid (15% 2 w/v, 0.4 ml) reagents were mixed with the supernatant. The samples were then placed in a boiling water bath. After cooling and centrifugation, the absorbances of supernatants were recorded by plate reader.

Bradford assay was performed to calculate the protein concentration in the cellular extracts and the MDA concentration was presented as nmol/mg protein based on a standard curve of MDA solution.

### 2.6 Comet assay

When the treatments of the cells, with or without linalool, were finished, the cells were harvested and suspended in low melting point agarose (1%, LMP in PBS). After that, they were placed on the glass slides, which were pre‐coated with normal melting agarose (1%, NMP in PBS). After the gel solidification of a third layer of LPM, the slides were exposed to lysis buffer at 4°C overnight. DNA unwinding was performed using an alkaline solution (300 mM NaOH, 1 mM Na_2_EDTA, pH > 13). After electrophoresis at 23 V and 300 mA at 4°C for 30 min, and removing the excess alkali and detergents (by washing with tris buffer, 0.4 M, pH 7.5), and staining with edithium bromide, the stained slides were examined by a fluorescence microscope (excitation filter 520–550 nm and barrier filter 580 nm) and photographed (Rajabian et al., 2016).

### 2.7 Western blot analysis of Bax, Bcl‐2, caspase‐3, and cacspase‐9 expressions

After the treatments, the total proteins were extracted by ice‐cold lysis buffer and with the addition of protease inhibitor. Equal amount of protein samples was loaded on SDS‐PAGE, fractionated and then transferred to PVDF. The membranes were blocked in non‐fat milk‐TBS (5% w/v), and then exposed to the indicated primary and HRP‐conjugated secondary antibodies, and visualized by chemiluminescence using an ECL reagent. Band densities were quantified by NIH Image J software (Rajabian et al., 2019).

### 2.8 Statistical analyses

All statistical analyses were carried out using the GraphPad Prism, version 8. All data were expressed as mean ± SEM. One‐way and two‐way analysis of variance was used to compare the differences between three or more groups. Tukey's test was used to compare the differences between the two groups. Differences were statistically indicated significant when *p*‐value was less than .05.

## 3 RESULTS

### 3.1 Cytotoxicity evaluation

MTT and comet assays were used in order to investigate the possible cytotoxic and genotoxic effects of linalool in PC12 cells, after 14 and 18 h exposure.

**FIGURE 1:**
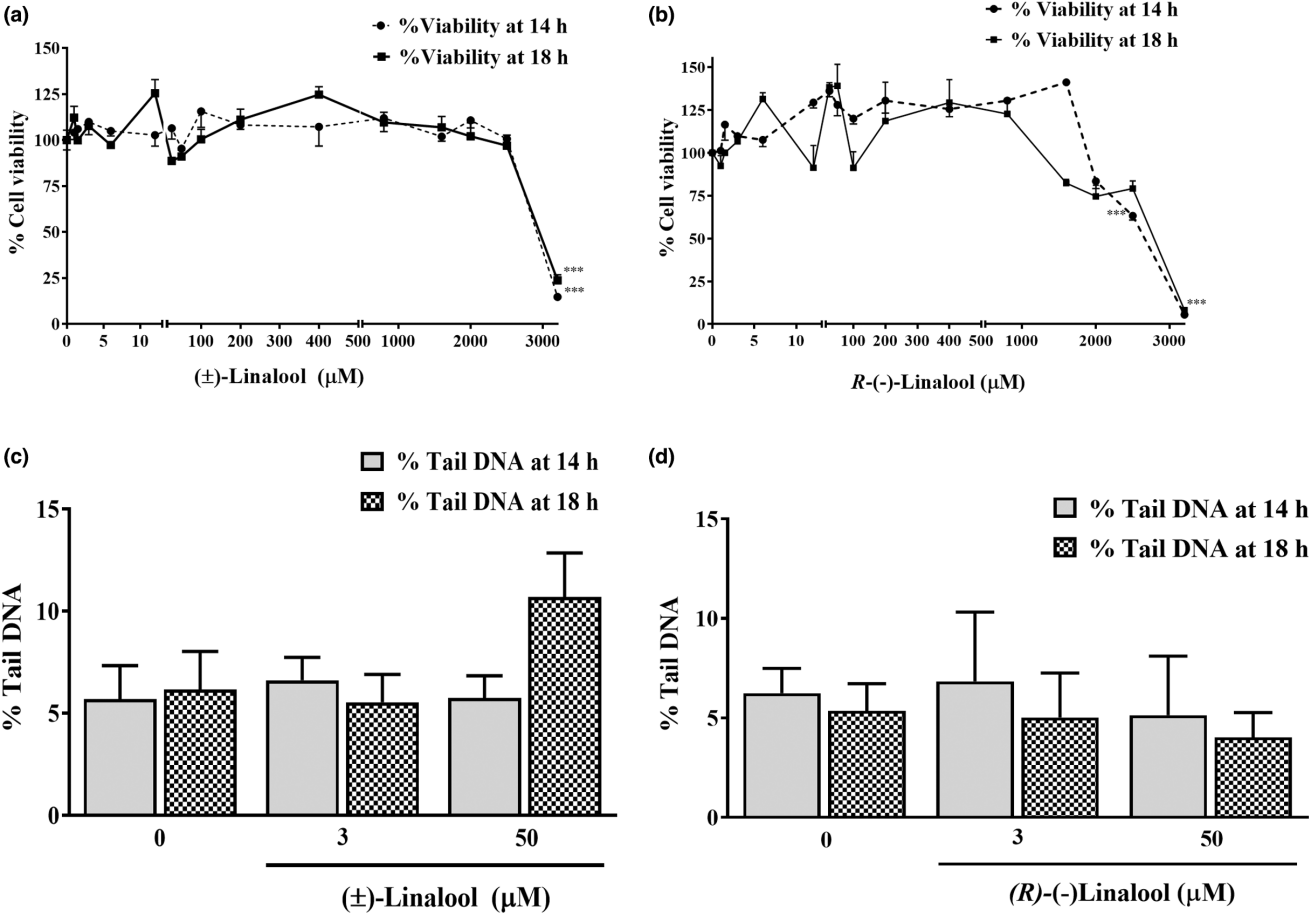
Effects of linalool on the viability (a and b) and the extent of DNA damage (c and d) in PC12 cells. The cells were incubated with (±) linalool (a and c) and (*R)*‐(−) linalool (b and d) at various concentrations for 14 and 18 h. The cells without any treatment were used as the control. The cell viability was determined via the MTT assay. The percent of DNA in the comet tail (% tail DNA) was estimated as an index for the extent of DNA damage. Data were presented as mean ± SEM. ^***^ *p* < .001 vs. control group.

Figure 1a,b shows the concentration‐response curve of (±) linalool and *(R)*‐(−) linalool. The cell viability significantly decreased by 85.3% and 76.2% after 14 and 18 h exposure to 3200 μM of (±) linalool (*p* < .001). Similarly, 94.5% and 92% decrease in the cell variability was observed after 14 and 18 h exposure to 3200 μM *(R)*‐(−) linalool, respectively (*p* < .001). Meanwhile, 14 h incubation of the cells with 2500 μM of *(R)*‐(−)‐linalool induced 36.8% (*p* < .001) decrease in the cell viability. Compared to the untreated cells, no significant change in viability was observed in the cells incubated with concentrations less than 3200 and 2600 μM of (±) linalool and *(R)*‐(−) linalool, respectively.

The concentrations inducing 50% cell growth inhibition (IC_50_ values) in PC12 cells following 14 and 18 h incubation with (±) linalool were 5440 and 2700 μM, as well as 2600 and 3040 μM following 14 and 18 h incubation with *R*‐(−)‐linalool, respectively (Figure 1).

The cell viability was above 90% when the cells were treated with concentrations below 100 μM; therefore, the three concentrations including 25, 50, and 100 μM were selected for the subsequent experiments.

As shown in Figure 1c,d, DNA damage of the PC12 cells increased after 14 or 18 h treatment with different concentrations of (±) linalool and *(R)*‐(−) linalool (3, 50, and 1600 μM). The DNA damage was measured in terms of DNA percentage in the comet tail (% tail DNA). Only the cells exposed to the highest concentration of (±) linalool (1600 μM) showed a significant increase (*p* < .05) in DNA damage compared to the control. After incubation of the cells with 1600 μM of (±) linalool for 14 and 18 h, DNA fragmentation significantly increased (21.36 ± 3.1% and 27.64 ± 2.3% *p* < .001). Similarly, incubation of the cells with *(R)*‐(−) linalool at 1600 μM for 14 and 18 h caused a significant DNA fragmentation to 15.2 ± 1.6% and 21.3 ± 2%, respectively, when compared with the untreated group (5.7 ± 1.6% and 6.2 ± 1.8% DNA in tail).

### 3.2 Linalool protected PC12 cells against OGD/R‐induced injury

**FIGURE 2:**
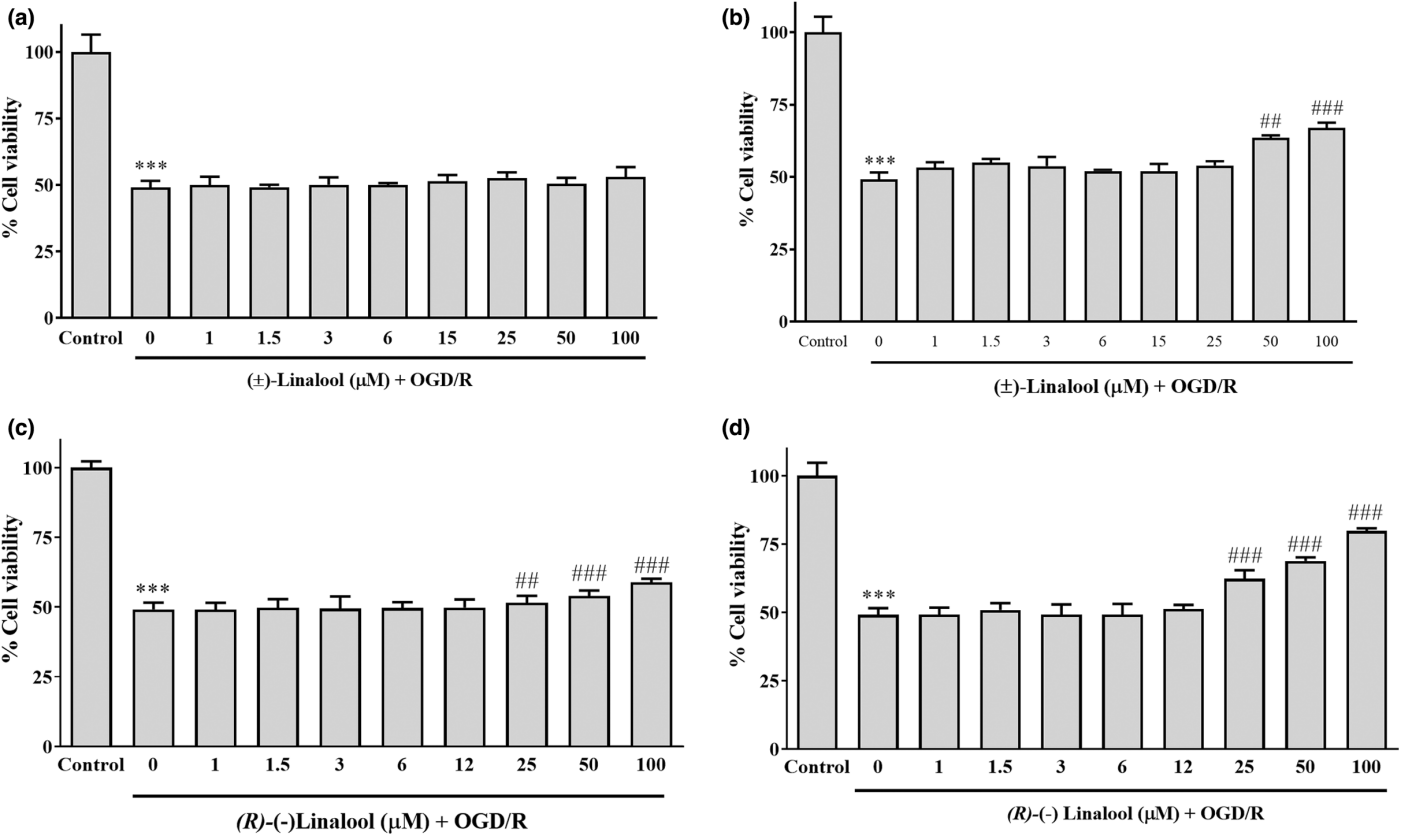
Effect of linalool on the viability of ischemic‐injured PC12 cells. The cells were pretreated with (±) linalool and (*R*)‐(−) linalool (1–100 μM;) for 2 (a and c, respectively) and 6 h (b and d, respectively) and then subjected to 12 h of oxygen–glucose‐serum deprivation and reoxygenation (OGD/R) condition. The cell viability was determined via the MTT assay. Data were presented as mean ± SEM., ***p < .001, **p < .01 vs. control group, ^##^ *p* < .001, ^###^ *p* < .01 vs. OGD/R group.

The cells cultured under OGD/R condition for 12 h exhibited a significant decrease in viability (Figure 2) to 49.11 ± 2.4% (*p* < .001). In contrast, pre‐treatment of the cells with 1–100 μM of (±) linalool for 6 h significantly increased the survival of OGD‐exposed cells to 63.5 ± 1% (50 μM, *p* < .01) and 67 ± 1.9% (100 μM, *p* < .001), respectively. However, in the cells pre‐treated with (±) linalool for 2 h prior to OGD insult, no improvement in the viability was observed. The significant differences between 2 and 6 h pre‐incubation with (±) linalool, at the above‐mentioned concentrations were also observed (*p* < .001).

On the other hand, incubation of the cells with (*R*)‐(−) linalool over the range from 1 to 100 μM for 2 h before OGD, improved the cellular viability to 51 ± 2.5% (25 μM, *p* < .01), 54 ± 2% (50 μM, *p* < .001) and 58 ± 1.3% (100 μM, *p* < .001), respectively. Treatment with *(R)*‐(−) linalool over a similar concentration range (1 to 100 μM), for 6 h before OGD also improved the cell survival to 62 ± 3.1% (25 μM, *p* < .001), 68.9 ± 1.3% (50 μM, *p* < .001) and 79.8 ± 1% (100 μM, *p* < .001), respectively. There was not any significant difference between 2 and 6 h pre‐incubation (*p* > .05).

### 3.3 Linalool suppressed OGD/R‐induced ROS generation in PC12 cells

**FIGURE 3:**
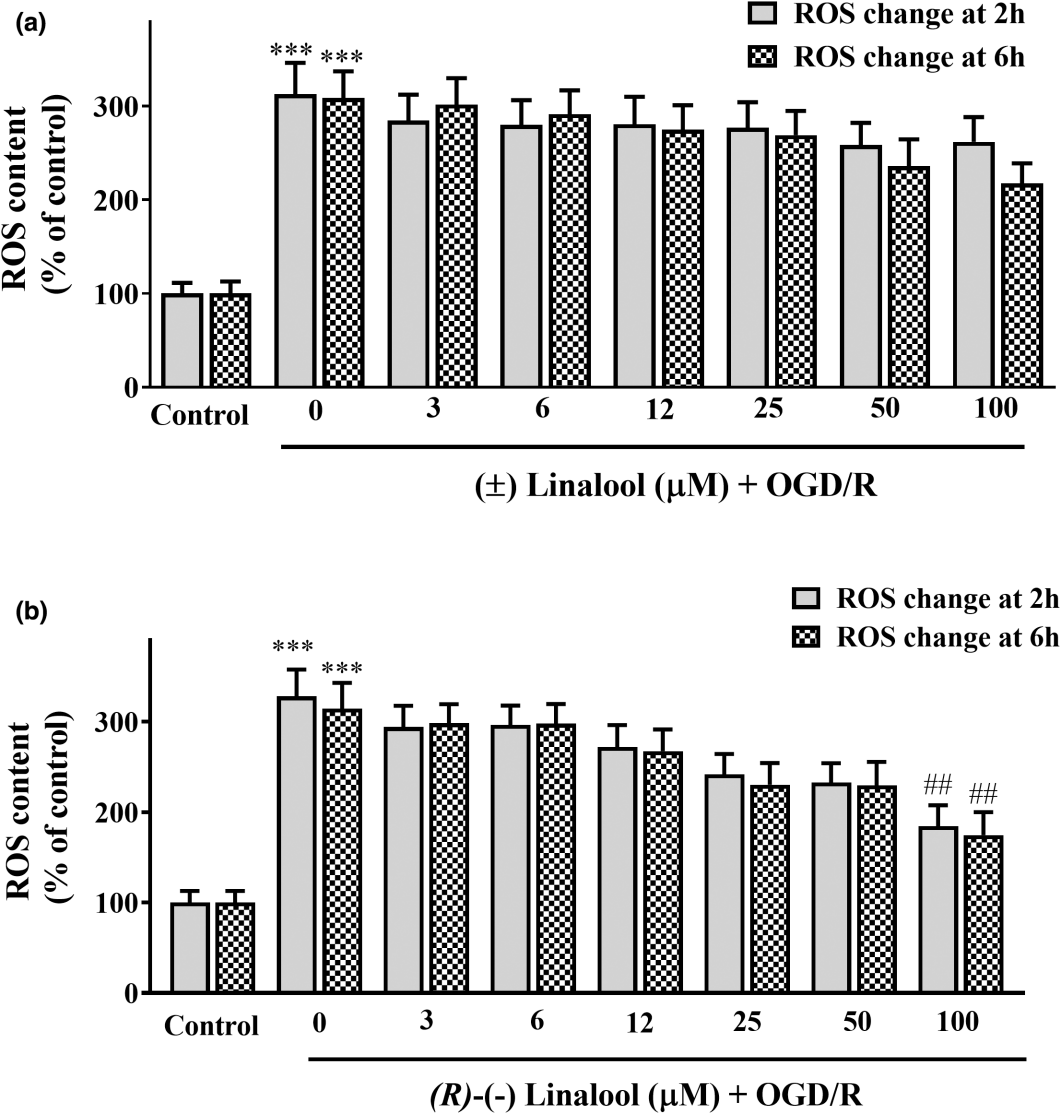
Effect of linalool on ROS production in the ischemic‐injured PC12 cells. The cells were preincubated with various concentrations of (±) linalool (a) and (*R)*‐(−) linalool (b) for 2 and 6 h and then subjected to 12 h of oxygen–glucose‐serum deprivation and reoxygenation (OGD/R) condition. The intracellular ROS was estimated via DCF fluorescence intensity. The cells without any treatment were used as the control. Data were presented as mean ± SEM. ***p < .01 vs. control group, ^##^ *p* < .01 vs. OGD/R group.

The results showed that OGD/R was associated with a remarkable increase (328 ± 29.5%, *p* < .001) in intracellular ROS generation which decreased by (±) linalool (Figure 3). However, ROS generation was significantly inhibited in the cells pre‐incubated with 100 μM of *(R)*‐(−) linalool to 184.3 ± 23.3% (*p* < .01) following 2 h and to 174.5 ± 25.5% (*p* < .01) following 6 h. No significant difference was seen between 2 and 6 h pre‐incubation of the cells with racemic or *(R)*‐(−) linalool (Figure 3, *p* > .05).

### 3.4 Linalool decreased OGD/R‐induced MDA overproduction in PC12 cells

**FIGURE 4:**
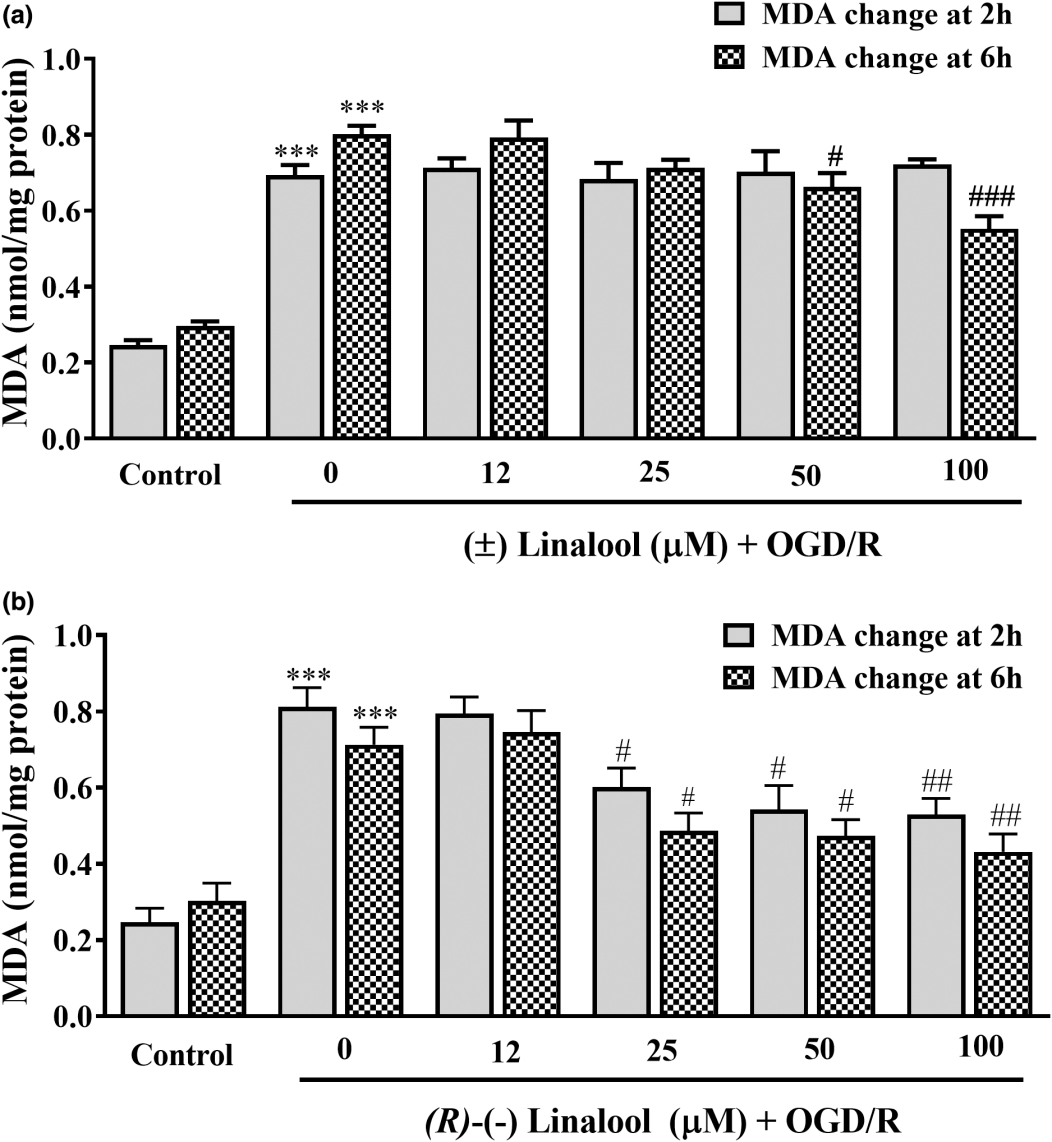
Effect of linalool on MDA production in the ischemic‐injured PC12 cells. The cells were preincubated with various concentrations of (±) linalool (A) and *(R)*‐(−) linalool (B) for 2 and 6 h and then subjected to 12 h of oxygen–glucose‐serum deprivation and reoxygenation (OGD/R) condition. The MDA level was determined via TBARS fluorescence intensity. The cells without any treatment were used as the control. Data were presented as mean ± SEM. ***p < .001 vs. control group, ^#^ *p* < .05, ^##^ *p* < .01, ^###^ *p* < .001 vs. OGD/R group.

OGD elevated MDA level to 0.81 ± 0.05 (nmol/mg protein), compared to the control group (0.24 ± 0.01 nmol/mg protein, *p* < .001). In contrast, MDA content was reduced in the cells pre‐treated with 50 and 100 μM of (±) linalool for 6 h to 0.66 ± 0.03 (*p* < .05) and 0.55 ± 0.03 (nmol/mg protein *p* < .001), respectively (Figure 4a). Moreover, a significant difference between 2 and 6 h pre‐incubation with (±) linalool at 50 μM was found (*p* < .05).

Pre‐treatment of the OGD‐injured cells with 25, 50, and 100 μM of *(R)*‐(−) linalool for 2 h reduced MDA content to 0.60 ± 0.05 (*p* < .05), 0.54 ± 0.06 (*p* < .05), and 0.53 ± 0.04 (*p* < .01, nmol/mg protein), respectively. Similarly, MDA level decreased to 0.48 ± 0.04 (*p* < .05), 0.47 ± 0.04 (*p* < .05), and 0.43 ± 0.04 (nmol/mg protein, *p* < .01), following exposure of the OGD‐injured cells to 25, 50, and 100 μM of *(R)*‐(−) linalool for 6 h, respectively (Figure 4b). A significant difference between 2 and 6 h pre‐incubation with *(R)*‐(−) linalool was also observed (Figure 4b, *p* < .001).

### 3.5 Linalool reduced OGD/R‐induced DNA injury in PC12 cells

**FIGURE 5:**
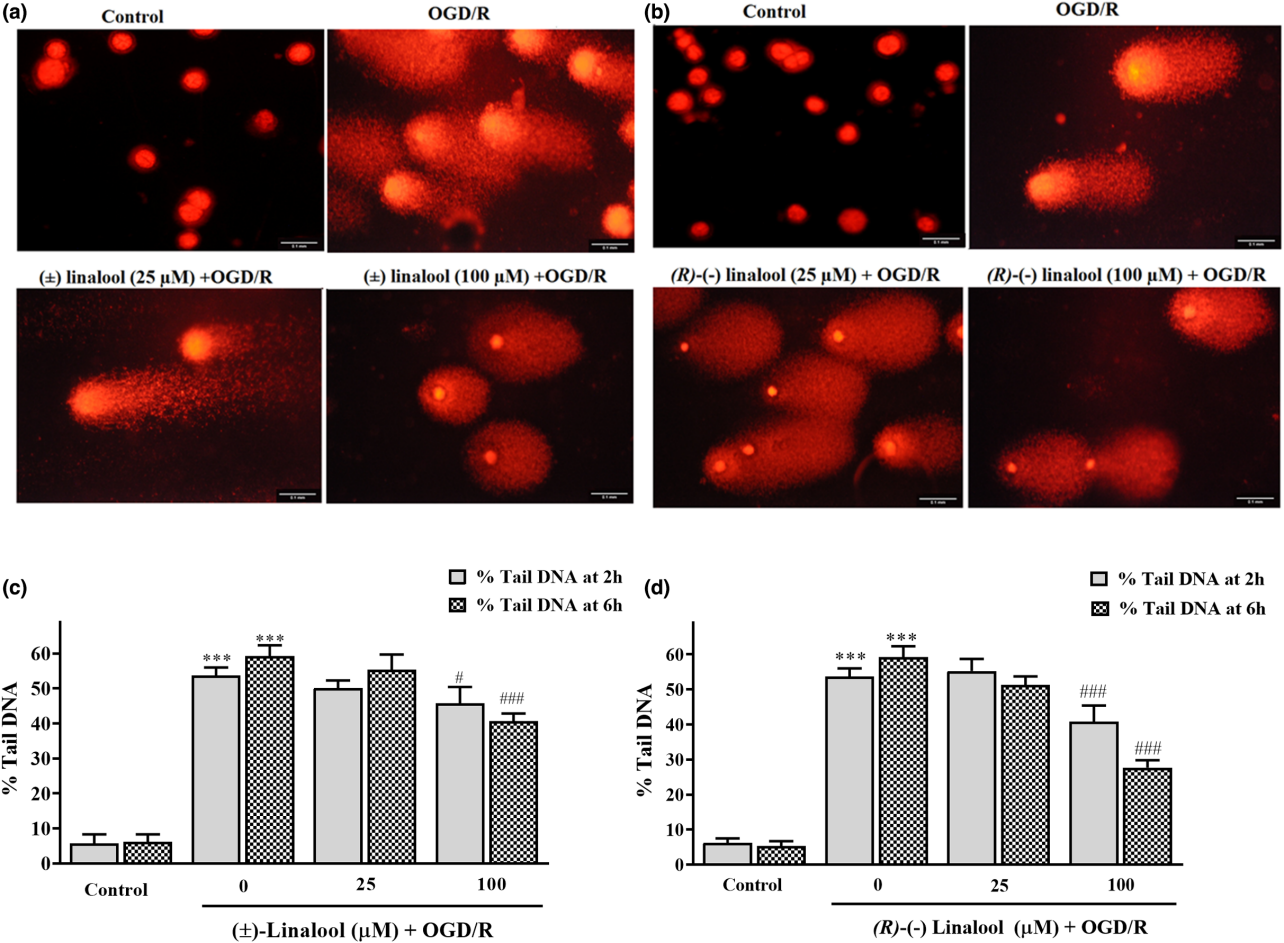
Effect of linalool on DNA damage in the ischemic‐injured PC12 cells. (a and b) representative comet images of the ischemic‐injured cells treated with (±) linalool and *(R)*‐(−) linalool. The cells were preincubated with different concentrations (25, 100 μM) of (±) linalool (c) and (*R)*‐(−) linalool (d) for 2 and 6 h and then subjected to 12 h oxygen–glucose‐serum deprivation and reoxygenation (OGD/R) condition. Scale bars indicate 0.1 mm. The percent of DNA in the comet tail (% tail DNA) was estimated as an index for the extent of DNA damage. ***p < .001 vs. control group, ^#^ *p* < .05, ^###^ *p* < .001 vs. OGD/R group.

Pre‐treatment with 100 μM of (*R*)‐(−) linalool for 2 h and 6 h significantly reduced OGD‐induced DNA injury, compared to those seen in OGD group (Figure 5c,d, 40.74 ± 4.7% and 27.7 ± 2.1% vs. 59.17 ± 3.1%, respectively). At the same concentration of (±) linalool (100 μM), a reduction of DNA fragmentation was achieved after 2 h and 6 h incubation (45.7 ± 4.7% (*p* < 0.05); 40.7 ± 2.1% (*p* < .001) vs. 59.17 ± 3.1%, respectively) (Figure 5a,b).

### 3.6 Linalool counteracted apoptotic cell death via caspase‐9/3 pathway in OGD/R‐injured PC12 cells

**FIGURE 6:**
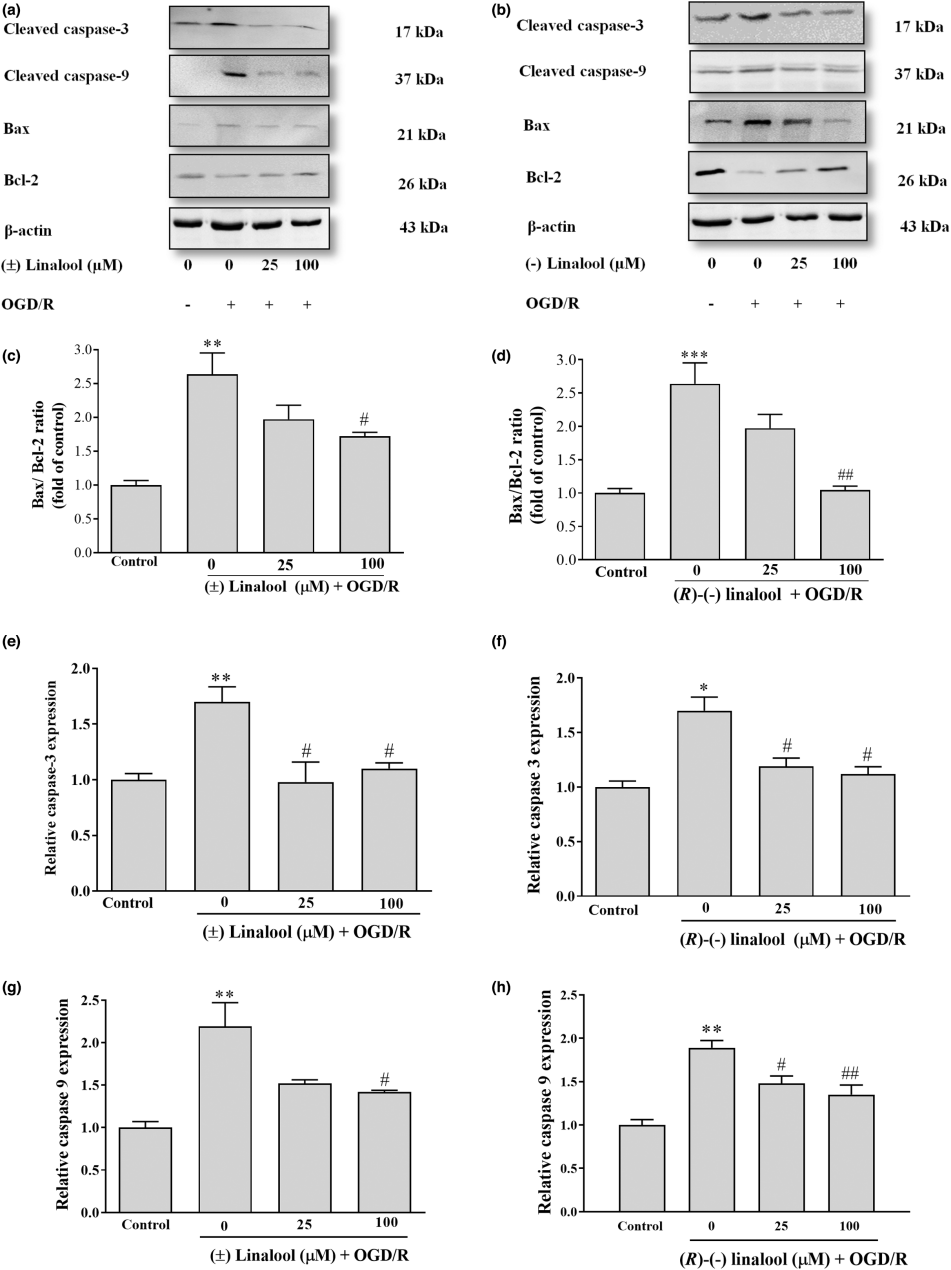
Effect of linalool on expression of apoptosis‐related proteins in the ischemic‐injured PC12 cells. The cells were preincubated with various concentrations (25 and 100 μM) of (±) linalool and (*R*)‐(−) linalool for 6 h and then subjected to 12 h of oxygen–glucose‐serum deprivation and reoxygenation (OGD/R) condition. (a and b) the representative blots of Bcl‐2, Bax, Caspase‐9, Caspase‐3, and β‐Actin proteins. (c–h) quantitative analysis of protein levels shown. **p < .01 and ***p < .001 vs. control group, ^#^ *p* < .05, ^##^ *p* < .01 vs. OGD/R group.

Compared to the control group, OGD insult reduced Bax/Bcl‐2 ratio (72.63 ± 0.3 vs. 1 ± 0.06, *p* < .001) and also elevated expression of cleaved‐caspase‐3 (1.7 ± 0.20 vs. 1 ± 0.02, *p* < .001) and caspase‐9 (1.6 ± 0.05 vs. 0.1 ± 0.01, *p* < .01) (Figure 6).

Pre‐incubation with (±) linalool and *(R)*‐(−) linalool at 100 μM remarkably mitigated the changes in the ratio of Bax/Bcl‐2 to 1.7 ± 0.06 (*p* < .05) and 1.04 ± 0.06 (*p* < .01), and attenuated the levels of cleaved‐caspase‐3 (1.1 ± 0.05 and 1.1 ± 0.06, *p* < .05, *p* < .01) and caspase‐9 (1.4 ± 0.2 and 1.3 ± 0.01, *p* < .05, *p* < .01), respectively (Figure 6).

## 4 DISCUSSION

**FIGURE 7:**
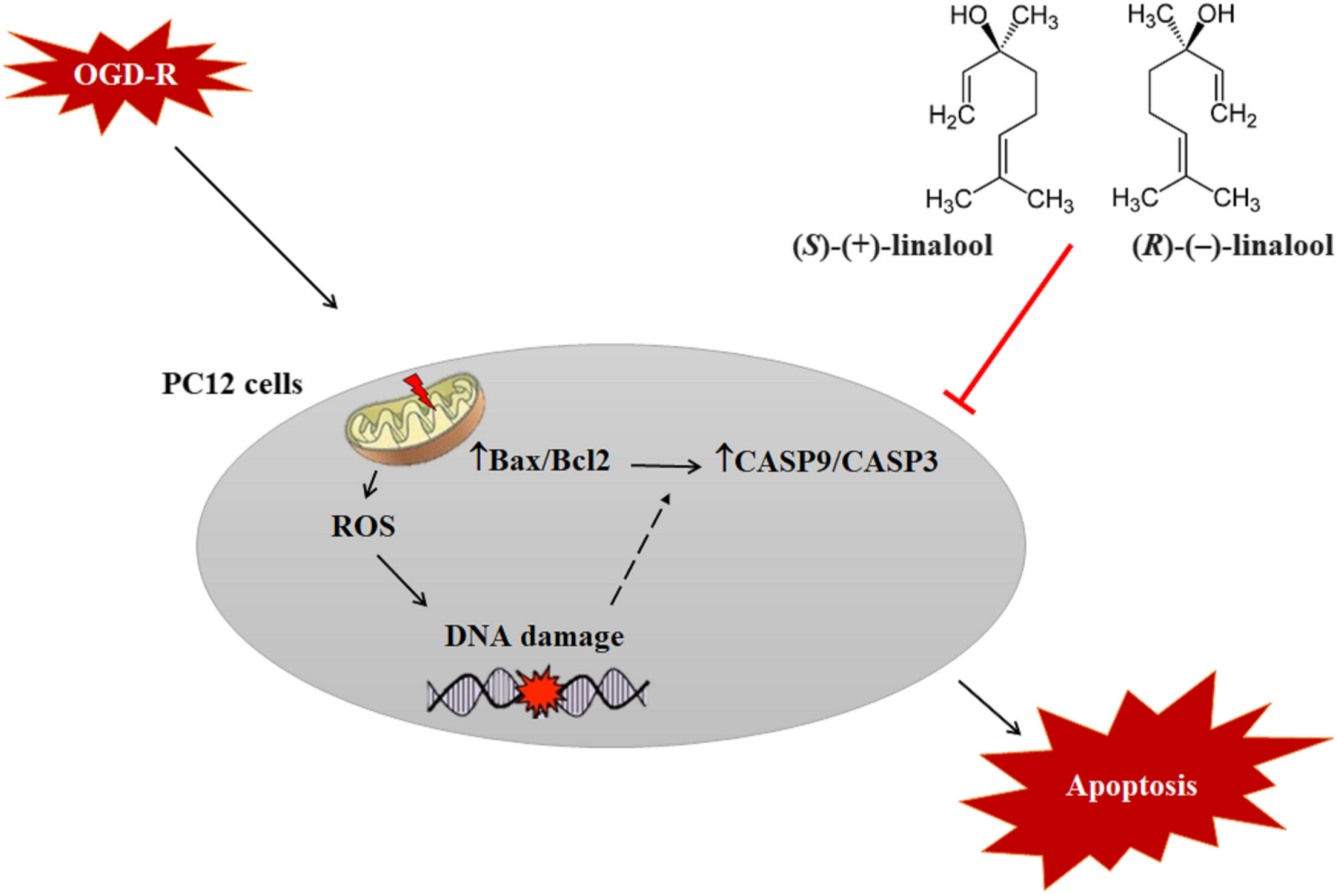
The schematic representation of the mode of actions of (±) linalool and *(R)*‐(−) linalool against PC12 cell death induced by oxygen–glucose‐serum deprivation and reoxygenation (OGD/R).

The main finding of the present study is that linalool significantly improved the PC12 cell survival following ischemia/reoxygenation injury. Linalool significantly inhibited OGD‐induced cytotoxicity by attenuating ROS overgeneration, lipid peroxidation, and oxidative DNA damage. The resulting oxidative stress‐associated apoptosis was also inhibited by linalool, via downregulations of Bax/Bcl‐2, caspase‐3, and caspase‐9 (Figure 7). While (*R*)‐(−) linalool had better influence on the values of the parameter of interest, but no significant difference was observed between two compounds.

The possible cytotoxicity of (±) linalool and *(R)*‐(−) linalool were also evaluated. Among the different concentrations analyzed, only 2500 and 3200 μM of *(R)*‐(−) linalool as well as, 3200 μM of (±) linalool showed cytotoxic potential, which were in agreement with previously reported data (Alinejad et al., 2013; Kim et al., 2015; Park et al., 2016).

Ischemia is associated with redox imbalance which results in oxidative stress and brain cell apoptosis (Rodríguez‐González et al., 2016). It was reported that oxidative stress causes irreversible damage in DNA and other macromolecules (Ge et al., 2018). In the present study, OGD in vitro model was used to mimic the pathological process of ischemia/reperfusion injury, as established previously, and to evaluate the neuroprotective potential of linalool on OGD‐induced cell injury.

In line with the previous studies (Khazdair et al., 2015; Yu et al., 2008), we found that excessive amounts of ROS can be generated in PC12 cells under OGD/R condition. Moreover, ROS can promote peroxidation of polyunsaturated fatty acids as indicated by increased malondialdehyde (MDA) production in our study.

Recent reports indicated that linalool protected neuronal cells from oxidative injury in the animal models of cognitive deficit induced by D‐galactose, AlCl3, and amyloid beta (Aβ)1–40 (Xu et al., 2017b). (*R*)‐(−) Linalool also counteracted acrylamide induced‐toxicity in brain tissue (Mehri et al., 2015). Free radical scavenging, attenuating lipid peroxidation, and improving the antioxidant capacity of hippocampus by linalool contributed to the neuroprotective effects (Xu et al., 2017b). (*R*)‐(−)‐linalool reduced H_2_O_2_‐induced oxidative stress in PC12 cells via decreasing of ROS, LDH, and apoptosis (Migheli et al., 2021). In addition, the anti‐oxidant activities of linalool have been reported in other cells such as HepG2 (Erdogan & Ozkan, 2017). Linalool decreased oxidative stress, lipid peroxidation, and ROS formation in human lymphocytes following benzene toxicity (Salimi et al., 2021). Linalool also attenuated benzene‐induced oxidative toxicity in rat liver by reduction of advanced oxidation protein products and MDA production, with concomitant elevation of superoxide dismutase (SOD), catalase (CAT), and glutathione (GSH) contents (Ola & Sofolahan, 2021). Linalool improved renal function following ischemia–reperfusion injury by mitigation of oxidative damage and restoring the activities of anti‐oxidant enzymes (Golmohammadi et al., 2021). Linalool also decreased doxorubicin‐induced renal injury by attenuation of oxidative stress and modulation of anti‐oxidant enzymes such as SOD and CAT (Altinoz et al., 2021). Also, linalool exerted protective effects against excitotoxic cell injury mediated by NMDA in the hippocampal slices (Airao et al., 2021; Sabogal‐Guáqueta et al., 2019). In vivo and in vitro investigations have demonstrated the antagonistic activity of (±) linalool on glutamatergic NMDA receptors. Therefore, linalool seems to modulate glutamatergic neurotransmission (Aprotosoaie et al., 2014). Linalool (100 μM) was found to exert antioxidant and anti‐apoptotic effects in glutamate‐mediated excitotoxity in HT‐29 cells (Sabogal‐Guáqueta et al., 2019). Excessive extracellular excitatory neurotransmitters, triggering oxidative stress, and apoptosis, are involved in neurodegeneration following cerebral ischemia (Chamorro et al., 2016).

The neuroprotective properties of linalool were also explored in some recent in vitro studies. Park et al. (2016) showed the efficacy of *(R)*‐(−) linalool in oxygen–glucose deprivation/re‐oxygenation (OGD/R) in cortical neuronal cells. *(R)*‐(−) linalool protected cortical neuronal cells against ischemic injury through free radical scavenging, restoring antioxidant defense systems, thereby attenuating oxidative injury.

In consistent with aforementioned evidences, we observed that linalool dose‐dependently suppressed OGD/R‐induced ROS and MDA overproductions. As reported in the previous investigations (Jung & Kwak, 2010; Xu et al., 2017a), enhanced cellular antioxidant capacity may be linked to the remarkable difference in the amount of lipid peroxidation between the OGD/R‐injured cells pre‐incubated with linalool for 2 or 6 h.

Our data demonstrated that *(R)*‐(−) linalool protected PC12 cells from OGD/R‐induced cell injury more efficiently than (±) linalool. Consistently, in a study conducted by Sugawara et al. (1998) it was found that the sedative activity of (±)‐linalool could be attributed to *(R)*‐(−) linalool but not (S)‐(+)‐enantiomer. *(R)*‐(−) Linalool was found to relieve some stress‐related physiological parameters of autonomous nervous system (heart rate and blood pressure, etc.). While, (S)‐(+)‐ isoform induced opposite effects including increased heart rate and blood pressure (Höferl et al., 2006). de Sousa et al. (2010) reported elongation of convulsions latency by linalool in animal model of pentylenetetrazol‐induced convulsions. However, the anticonvulsant effect of linalool racemate was more potent than its enantiomers. Two enantiomers showed similar qualitative anticonvulsant activities with different potencies.

Subsequent to OGD, ROS activate a cascade of deleterious processes which eventually promote neuronal cell death (Jin et al., 2018). Energy deprivation, mitochondrial dysfunction, and overproduction of free radicals can induce DNA damage which eventually results in cell apoptosis (Guo et al., 2013). Death signals following ischemia–reperfusion injury activate Bcl‐2 family members which leads to activation of the mitochondria‐dependent caspase signaling pathway (Mousavi et al., 2010; Shafaei‐Bajestani et al., 2014). Bax over‐expression can promote the apoptosis (Gao et al., 2016).

Our observations are also in agreement with previous studies revealed the neuroprotective effects of (−) linalool against OGD or H_2_O_2_‐stimulated neuronal injury. Linalool protected PC12 cells against H_2_O_2_ by attenuation of oxidative stress and apoptosis. Also, linalool inhibited inflammation and oxidative stress in cortical neuronal injury induced by OGD/R (Migheli et al., 2021; Park et al., 2016).

Studies have demonstrated that downregulation of Bcl‐2 caused decreased cell viability under ischemia–hypoxia condition. In cerebral ischemia, Bcl‐2 was considered as an indicator protein for nerve cell apoptosis (Ouyang & Giffard, 2004; Wei et al., 2005). Since Bcl‐2 possesses free radical scavenging activity, it is capable of suppressing peroxidation of lipid and DNA injury exerted by deleterious agents and initiators of apoptosis in PC12 cells (Jang & Surh, 2003). Previous studies have reported an increase in the expressions of Bax and cleaved‐caspases 3, 9 following cerebral ischemia (Gao et al., 2016; Tian et al., 2018).

As indicated in the previous studies, linalool could reduce rate of cardiomyocyte apoptotic death induced by isoproterenol, by suppressing Bax protein expression (Zheng et al., 2017). Similarly, Gunaseelan et al. (2017) showed that linalool blocked apoptosis of HDFa skin cells following UV‐B exposure by modulating of redox status and expressions of Bax and Bcl‐2 proteins. In the same way, down‐regulation of cleaved caspases‐3 and‐9 was suggested in the neuroprotective role of linalool (100 mg/kg) in Aβ‐induced neurotoxicity in rat hippocampus (Xu et al., 2017b).

## 5 CONCLUSION

The above‐mentioned results revealed the protective effects of linalool on neurons under ischemia. Linalool prevented OGD‐stimulated toxicity by mitigating ROS overgeneration, lipid peroxidation, and oxidative DNA damage. The resulting oxidative stress‐associated apoptosis was also inhibited by linalool via downregulation of Bax/Bcl‐2, caspase‐3, and caspase‐9. *(R)*‐(−) Linalool and (±) linalool have a similar profile of neuroprotective potential. While (*R*)‐(−) linalool had better influence on the values of the parameter of interest, but no significant difference was observed between two compounds.

## CONFLICT OF INTEREST

The authors declare that there is no conflict of interest.

## Data Availability

The data that support the findings of this study are available from the corresponding author upon reasonable request.
